# The Minimum Purchase Price policy in China and wheat production efficiency: a historical review, mechanisms of action, and policy implications

**DOI:** 10.3389/fnut.2025.1536002

**Published:** 2025-03-19

**Authors:** Yangyang Chen, Ming Cheng

**Affiliations:** ^1^College of Economics and Management, Zhejiang A&F University, Hangzhou, China; ^2^Zhejiang University of Water Resources and Electric Power, Hangzhou, China

**Keywords:** Minimum Purchase Price policy, wheat production efficiency, development history, mechanisms of action, grain yield

## Abstract

This study addresses challenges in China’s staple grain production efficiency by examining the impact of the Minimum Purchase Price policy (MPP) on wheat production efficiency from the perspective of price support. First, relevant literature on price intervention policies is collected, summarized, and organized. This paper reviews previous research findings on the effects of the Minimum Purchase Price policy, social welfare implications, pricing studies, and comparative analyses of wheat production efficiency. Second, the study outlines the development of the Minimum Purchase Price policy and wheat production efficiency, and theoretically analyzes the mechanism by which the policy influences production efficiency. Finally, four recommendations for enhancing grain policy and improving wheat production efficiency in China are proposed: a focus on balanced macroeconomic development and coordinated efficiency, accelerating the allocation of production factors to unlock wheat production’s potential, fostering a conducive external social environment for industrial integration, and optimizing the Minimum Purchase Price policy to maximize its benefits.

## 1 Introduction

The 2024 Central No. 1 Document prioritizes the advancement of the structural reform of agricultural supply and the enhancement of grain supply security. Increasing grain production is essential for addressing grain issues. The implementation and improvement of the Minimum Purchase Price policy are critical for ensuring grain security, achieving high wheat yields, and securing farmers’ income. As trade policy liberalization progresses, several major grain-producing provinces in China are caught in the paradox of “simultaneous growth of three metrics and enhancement of three benefits” and face the dilemma of “low grain prices harming farmers, and high rice prices harming consumers.” As a primary industry, agriculture forms the foundation for the development of other sectors. Economic growth in agriculture relies mainly on increasing input factors or improving efficiency; the former drives overall economic growth, whereas the latter enhances both the quantity and quality of growth. Thus, exploring the effects of the Minimum Purchase Price policy on wheat production efficiency in the context of high-quality and high-standard agricultural development is of practical significance.

In 2023, China maintained stable grain production for the ninth consecutive year, surpassing 1.3 trillion *jin* (unit of weight, 1 jin equals to 500 g), with a total national grain output of 1,390.82 billion *jin*, a 1.3% increase over the previous year. Nevertheless, this growth rate was 0.3% lower than the average annual growth rate over the past two decades, indicating a decline in the momentum of grain production growth. As economic and natural conditions evolve, the factors driving agricultural production change. Relying on increased input factors to boost the yield per unit area is becoming unsustainable. Promoting continuous growth in grain productivity and achieving intensive agricultural production are crucial for transitioning from high-speed to high-efficiency economic growth. In response to these challenges, the state implemented a series of agricultural subsidy policies. Among these, the Minimum Purchase Price policy introduced in 2004 was a price-control measure targeting China’s major staple grains. It aims to protect farmers’ enthusiasm for grain cultivation, safeguard agricultural income for grain-producing households, and ensure that grain production meets market demand.

Since the implementation of the Minimum Purchase Price policy, evaluations of its effects have been inconsistent. This policy has acted as a “double-edged sword.” Its benefits include increased grain production, regulated rational circulation in the grain market, and stimulated enthusiasm for farm production. It has also contributed to reasonable grain prices, buffering input cost fluctuations and ensuring farmers’ income from grain cultivation. Additionally, it has improved the ecological environment for grain cultivation, enhanced overall social welfare, and promoted the cyclical development of modern agriculture. However, with changes in the domestic and international economic environments, the drawbacks of this “price subsidy combined” policy have become apparent. First, the grain purchase price distorts the market price formation mechanism, leading to increased basic costs for enterprises using grain as a production material, whereas farmers’ income from grain cultivation has not fundamentally improved. Second, price inversion between domestic and international grain markets has trapped China in the paradox of storing grain on a large scale while simultaneously importing large quantities of low-priced foreign grain, resulting in the coexistence of high domestic grain prices, high import volumes, and high stockpiles. Third, the continuous initiation of state procurement in major production areas has severely affected the grain market’s autonomy, hindering the market’s self-regulation mechanism and adversely affecting the optimization of supply-side structural reforms.

Furthermore, over time and with regional economic development, grain production efficiency may improve owing to more rational land rotation practices, higher-quality input factors, advanced technological innovations, and more efficient mechanization. These factors influence the net evaluation of policy effectiveness. In particular, due to the policy’s impact on increasing input factors, grain production may encounter diminishing marginal returns. Therefore, this study systematically reviewed the development of the Minimum Purchase Price policy since the founding of the People’s Republic of China and explored the mechanisms by which this policy affects wheat production efficiency. The goal was to supplement the existing research and provide a reference for the formulation of national macro-control policies.

## 2 Minimum Purchase Price policy and wheat production efficiency: research methodology and research Frontiers

### 2.1 Research method and data sources

This paper is a literature review aimed at evaluating the impact of China’s Minimum Purchase Price policy on wheat production efficiency. Relevant domestic and international research on wheat production efficiency was systematically reviewed, focusing on the research methods, data sources, and key findings. A qualitative analysis approach was employed to compare the results of different studies, summarizing both commonalities and divergences in the policy’s effects. The structure of the paper is as follows: first, the background and implementation of the Minimum Purchase Price policy are discussed; second, a review of the relevant literature is provided; third, the impact of the policy on wheat production efficiency and its underlying mechanisms are summarized; and finally, policy recommendations are proposed. It is important to note that due to differences in research methods and data sources, inconsistencies exist in the existing studies, which may affect a comprehensive assessment of the policy’s overall impact. Agricultural data used in this study are sourced from the annual official statistics of the National Bureau of Statistics of China, while minimum purchase price data are provided by the National Development and Reform Commission and other relevant departments.

### 2.2 Minimum Purchase Price policy

The Minimum Purchase Price policy, a price-support measure for China’s major staple grains, plays a crucial role in achieving the overall objectives of agricultural policy. Academic research on this policy mainly focuses on two aspects: its social welfare effects and pricing studies related to the minimum purchase price. As for the social welfare effects, agricultural price support policies essentially redistribute benefits among various market participants. This redistribution can enhance fairness in welfare distribution but also result in unnecessary global welfare losses and decreased efficiency. Over time, the policy’s impact on social welfare has varied. Initially, it created a win-win situation for all participants (consumers, producers, private grain enterprises, the government, etc.) and overall social benefits. However, in the later stages, some participants (such as consumers and private grain enterprises) and overall social welfare suffered, revealing the dual nature of the policy. Comparing the welfare changes before and after policy implementation, it was found that grain producers’ welfare increased, grain consumers’ welfare decreased, grain processing enterprises’ welfare was harmed, government welfare weakened, and overall social welfare declined. The main reason for this is that consumers bear part of the government’s transfer expenditure, reducing their welfare. The grain price control system entails significant institutional costs, and only by effectively reducing these costs can grain price support policies truly enhance global total welfare effects. Anderson used a global economic model to evaluate the nominal assistance rate trends of 75 countries from 1955 to 2007, revealing that since 1984, structural reforms have continuously reduced price distortions, improving global economic welfare and social security while reducing inequality and poverty (1). Attanasio et al. analyzed the impact of price increases on welfare, noting that although price subsidy policies prevent supply and demand fluctuations caused by price volatility, they distort prices (2). Wang and Wei assessed the impact of agricultural price-support policies on distribution and overall welfare (3). They discovered that following the implementation of the policy, the net welfare change in China’s domestic market was negative, characterized by an increase in producer surplus and a decrease in consumer surplus.

Regarding pricing studies related to the Minimum Purchase Price policy, the grain price management system has evolved from a non-market-based framework to one that aligns with the current socialist market economy in place since the founding of the People’s Republic of China. China’s grain price-control policies have undergone six major reforms: unified purchase and sale price, dual-track price system, open grain prices, return to the dual-track price system, policy protection price, and minimum purchase price. Today, the minimum purchase price has become a “weathervane” for the grain market, increasingly characterized as a “policy market.” The domestic grain market has shifted from supply–demand tension to periodic surpluses for certain varieties, exacerbating the conflict between market openness and policy regulation in China. The first to notice the impact of grain price regulation policies on price fluctuations were Segal and Hoffman, who demonstrated the positive effects of state regulation policies on grain prices during wartime. However, Shepherd was skeptical of this conclusion. Since then, academic debates have focused on the role of state regulation policies in grain price fluctuations. Proponents of price intervention policies such as Swaminathan and Vepa found that grain policies can help reduce price volatility (4). Using a general equilibrium model, Gohin and Zheng showed that lowering price support levels exposes risk-averse European farmers to further price fluctuations by capturing different sources of risk, farmers’ risk attitudes, and contingent markets (5). In particular, after achieving trade policy liberalization, price support policies help mitigate the negative impact of industrialized countries’ agricultural trade policies on agricultural production in developing countries, protecting farmers who rely on agricultural products for their livelihoods from the impact of surging imports. Conversely, proponents of free trade argue that using grain reserves to mitigate price fluctuations is ineffective. Most developing countries have adopted high-inventory and high-cost methods to regulate market supply and demand. Jha and Srinivasan found that the cost of stabilizing prices by increasing stockpiles in India was too high and advocated a more market-oriented approach to reducing prices (6). Brooks demonstrated that the spillover effects of international price support and protection policies are gradually weakening (7). Establishing policies for emerging economies not only helps eliminate trade distortions but also contributes to the construction of a multilateral trading system.

Grain price controls evolved alongside China’s economic development. Low grain prices can harm farmers, whereas excessively high prices can increase the burden on consumers. Domestically, opinions on grain price controls vary. Most scholars believe that given the current land-to-population tension in China, grain prices should be regulated. The sudden outbreak of COVID-19 in 2020 raised concerns about food security. Xie and Wang found a long-term equilibrium relationship between agricultural product price fluctuations and China’s grain production (8). Maintaining a reasonable minimum purchase price helps stabilize grain production and price fluctuations. Wang and Wei found that with increased soybean support policies, price transmission elasticity gradually decreased, reducing domestic price volatility and increasing global grain price fluctuations (3). Additionally, Tao argued that although the policy of “supporting low and stabilizing high” is not directly reflected in futures prices, economic policy uncertainty significantly impacts grain futures’ price volatility (9).

### 2.3 Wheat production efficiency

After conducting a systematic review of the literature on grain production efficiency, it is evident that research on the efficiency of major staple crops is relatively well-developed. From a macro perspective, Covaci and Sojková used farm panel data from 2000 to 2004 to explain wheat production efficiency and productivity development in Slovakia (10). Balcombe et al. used Bayesian methods to examine changes in the technical efficiency of rice production in Bangladesh (11). Rakotoarisoa employed a dynamic panel model to compare rice productivity across 33 rice-producing countries and found that agricultural policies widened the yield gap between rich and poor countries (12). El-Rasoul et al. used a production function estimation model to calculate the total factor productivity of wheat crops in Egypt and revealed that wheat efficiency growth was insignificant (13). Mahmood et al. analyzed the economic benefits of wheat farmers in Pakistan and showed that participation in training improves the economic benefits for wheat growers (14). Aslam et al. rated the average technical productivity of rice and wheat production in Pakistan, India, and China and found that the average technical productivity calculated by the CCR model was 0.87 and that by SBM was 0.86, both significantly lower than the ideal values of the original DEA (15). Rachman et al. used logit regression to study the impact of technical efficiency on food security in rice planting, finding very low technical efficiency in East Java Province. From a micro perspective (16). Krasachat measured the technical efficiency of 74 grain farmers in Thailand and analyzed the related differences (17). Loke et al. randomly selected 124 private farms engaged in wheat production in the Samarkand region, and under the assumptions of constant returns to scale and variable returns to scale, measured average technical efficiency at 0.79 and 0.82, respectively, indicating considerable potential for yield improvement (18). The key determinants of technical efficiency include farmers’ age, agricultural education, soil fertility, and seed quality. Awal and Awudu examined data from 412 small farmers in northern Ghana to explore the role of farmer groups in improving rice yields and technical efficiency (19).

Furthermore, academic research has progressed to the stage of emphasizing the protection of agricultural production. Achieving global food security and agricultural sustainability are the dual goals of sustainable development (20). Costanzo and Bàrberi reviewed the application of agricultural biodiversity in wheat production, suggesting that biodiversity strategies in wheat cultivation can enhance the sustainability of farming systems (21). Morteza et al. used the standard ISO life cycle assessment method to compare the environmental impacts of different wheat production systems, aiming to identify cultivation systems that minimize environmental pollution (22). Gliessman argued that common food policies can inspire the global development of sustainable food systems (23). Liu et al. developed a decision satisfaction evaluation model based on the analytic hierarchy process and fuzzy comprehensive evaluation, confirming the mechanism by which government control of key staple grain market circulation prices supports the sustainable development of the national grain industry (24). Lankoski and Thiem combined productivity and environmental sustainability by empirically analyzing the impact of agricultural support policies on sustainable productivity (25).

## 3 Historical review

Since 1949, China’s grain procurement pricing policy has undergone four distinct phases: the free-trade stage (1949–1953), planned procurement stage (1953–1985), dual-track procurement stage (1985–1998), and market-oriented procurement stage (1998 to present). By 2003, grain production had gradually declined, and the sown area had continuously decreased. In response, the central government began establishing a major grain price support policy system in 2004, including minimum procurement price policies for wheat, early indica rice, mid-to-late indica rice, japonica rice, and temporary storage policies for corn and soybeans. These policies were initially implemented as pilot projects in major grain-producing provinces and underwent continuous optimization and reform over the next decade. In 2014, the temporary storage policy for soybeans was replaced with a target price subsidy policy. Subsequently, in 2016 and 2017, the “separation of price and subsidy” policy replaced the temporary storage policy for corn and the target price subsidy policy for soybeans, respectively. National policy adjustments are closely linked to the effectiveness of policy implementations. China is gradually shifting from policy-based grain procurement to market-based procurement, using the minimum procurement price as a regulatory tool.

The Minimum Purchase Price policy was introduced in 2004 and initially targeted rice. However, owing to the higher grain prices that year, the policy was not implemented until 2005. Wheat from 2006 was included in this study. Consequently, the Minimum Purchase Price policy has undergone three developmental stages since its implementation.

Initial stage: Low-grain inventory (2004–2006). In 2003, China’s total grain output was 430.6953 million tons, the lowest in history. Despite entering the middle-income stage of economic and social development, significant urban–rural income disparities have prompted the massive migration of rural laborers to urban areas for industrial jobs. Ensuring agricultural stability and food security has become a priority, with increasing agricultural income for farmers being the primary focus. In 2004, the government piloted the minimum purchase price protection policy. This policy uses an indirect subsidy method that combines price and subsidies to reduce policy-driven grain regulation and enhance market mechanisms.

Full implementation stage: Consecutive bumper harvests (2007–2012). From 2004 onward, China experienced consecutive bumper harvests. To prevent low grain prices from harming farmers and stabilize grain production, the government implemented a comprehensive Minimum Purchase Price policy. Stimulated by this policy, China’s grain production rate has consistently increased, and grain output motivation has remained strong. Farmers’ enthusiasm reached unprecedented levels, leading to the expansion of staple grain planting areas and a significant increase in agricultural income. Additionally, this policy stabilized the domestic grain market and prices, mitigating the impact of international grain price fluctuations on China’s grain market, thus improving social stability. With rapidly increasing agricultural production costs, the minimum purchase price continues to increase.

**Figure 1:**
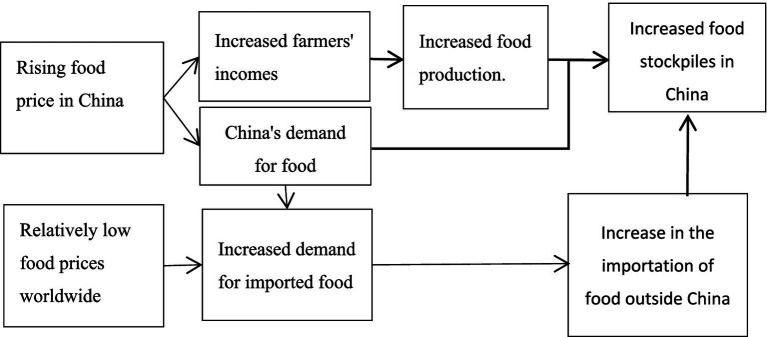
Internal logic diagram of “simultaneous growth of three metrics and enhancement of three benefits”.

Challenge stage: Weakened bottom-line support (2012–present). By 2015, China had achieved 12 consecutive years of growth in grain production. However, this success brought pressure to prevent grain prices from decreasing, gradually weakening the supporting role of the policy. The policy has led to high stock levels, increased imports, and elevated domestic prices, which, in turn, have adversely affected consumers. The price increases not only raise the cost of living for low-income households but also have the potential to exacerbate overall inflationary pressures, thereby affecting consumer purchasing power. At the same time, agricultural enterprises face significant cost pressures under price controls, which may lead to inefficiencies in resource allocation, thereby impacting profitability and long-term competitiveness. Furthermore, the increased import volumes under the policy could undermine domestic agricultural self-sufficiency, further distorting market prices. The market’s self-regulation mechanism started to falter, leading to numerous issues emerging during the later stages of policy implementation. The policy’s effectiveness in incentivizing production was negligible. On the one hand, economic volume, fiscal revenue, and resident income all increased simultaneously, leading to a situation in which domestic and international grain prices inverted. This inversion prompted enterprises to import large quantities of grain, reduced domestic demand, and forced the state to purchase more grain, thereby increasing storage pressure. However, producers, enterprises, and the state have encountered increasing pressures related to economic efficiency, social benefits, and ecological benefits. The minimum purchase price could no longer cover producers’ production costs; the cost of production for grain-based enterprises increased, and state storage costs rose daily. These challenges have necessitated reforms in the Minimum Purchase Price policy to correct excessive market intervention. Therefore, in 2016, the state lowered the minimum purchase price for the first time to establish a market-based pricing mechanism (Figure 1).

Table 1 illustrates the evolution of policy regarding the covered varieties and regions. Initially, a Minimum Purchase Price policy was implemented for early indica rice in major production areas. By 2006, the policy was expanded to include mid-to-late indica rice, japonica rice, and wheat. The scope of implementation also broadened, starting with the 4 provinces of Hunan, Hubei, Jiangxi, and Anhui and eventually encompassing 13 provinces.

**Table 1:** Implementation scope of the minimum purchase price policy.

Name	Policy introduction time	Area
Early long-grain nonglutinous rice	2004	HuNan, HuBei, TiangXi, AnHui
	2008	Guangxi
Late long-grain nonglutinous rice; Medium long-grain nonglutinous rice	2006	Hubei, HuNan, JiangXi, AnHui, Hei LongJiang, SiChuan, JiLin
	2008	JiangSu, LiaoNing, HeNan, GuangXi
Wheat	2006	Hebei, HeNan, ShanDong, HuBei, JiangSu, AnHui

**Figure 2:**
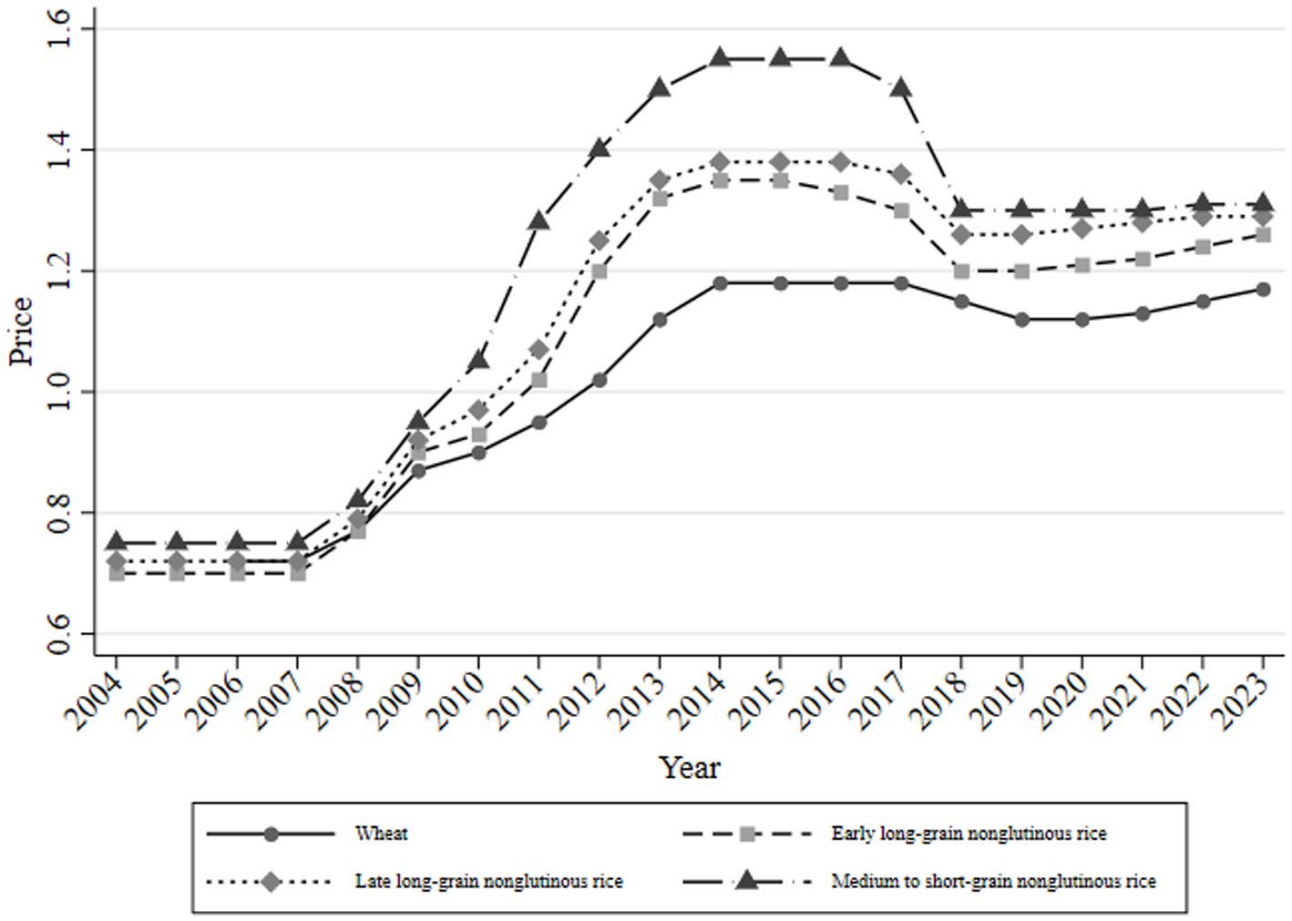
Changes in the minimum purchase price.

Figure 2 shows the price changes based on the minimum purchase price notifications for wheat, early indica, mid-to-late indica, and japonica rice. The annual minimum purchase price disclosed to the public before spring planting considers the grain supply–demand relationship, profit margins, production costs, natural disasters, and various macroeconomic factors. This announcement aimed to guide farmers’ planting decisions and adjust the following year’s grain production. Since the minimum purchase price policies for rice and wheat were introduced in 2004 and 2006, respectively, the prices have undergone several phases: “increase—stabilize—decrease—stabilize—increase.” In 2017, the minimum purchase price was lowered for the first time, indicating greater elasticity and flexibility in price controls under the supply-side reforms.

**Figure 3:**
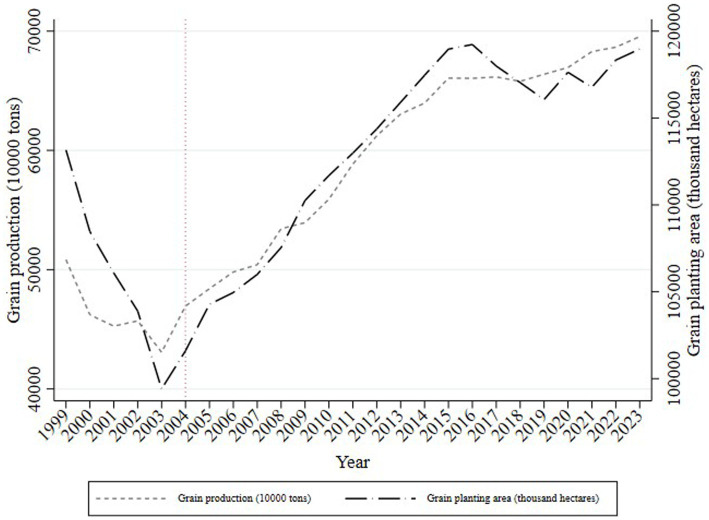
Changes in grain yield and planting area.

Figure 3 illustrates the overall V-shaped trend in grain output and planting area changes from 1999 to 2023. Before 2004, China’s grain production management was lax, which led to significant decreases in both grain output and planting area. After 2004, as the planting area increased, grain output grew accordingly, achieving 13 consecutive years of growth by 2015 with an average annual increase of 4.45%. The grain output growth of this period relied primarily on the straightforward expansion of the planting area. A series of pro-farmer policies introduced in 2004, such as direct grain subsidies, comprehensive agricultural input subsidies, and temporary storage policies, directly stimulated an increase in staple grain production.

However, from 2016 onward, despite a reduction in planting area, grain output remained stable, indicating reduced dependency on land for grain production. Other input factors have also begun to play decisive roles.

**Figure 4:**
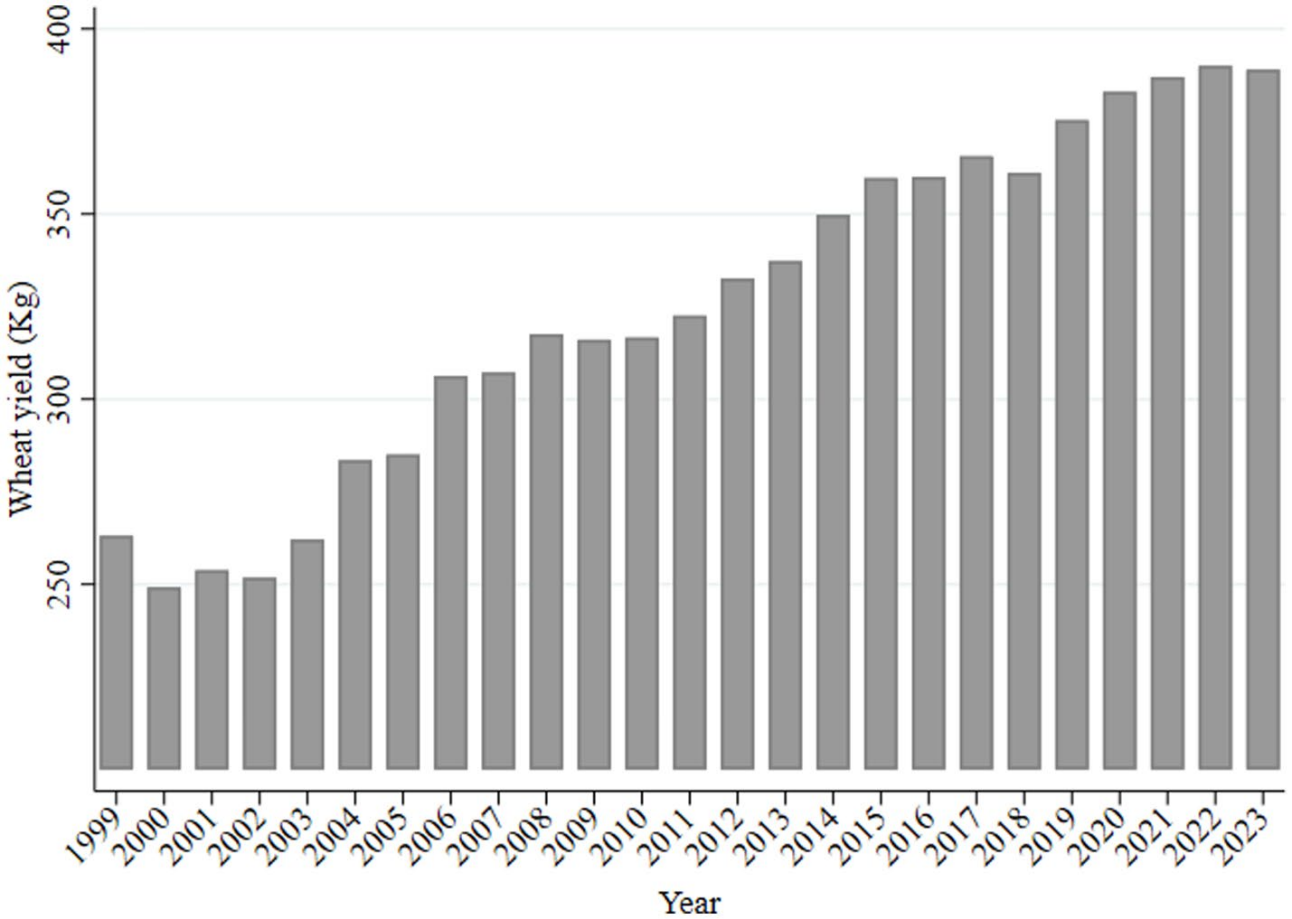
Variation in wheat yield per unit area.

The implementation of the Minimum Purchase Price policy for wheat in 2006 directly boosted the total wheat production to over 100 million tons. Total wheat production is closely related to yield per unit area, which more accurately reflects wheat productivity. Figures 4 shows the changes in wheat yield per unit area in the provinces in which the policy was implemented from 1999 to 2023, indicating an overall upward trend with fluctuations.

**Figure 5:**
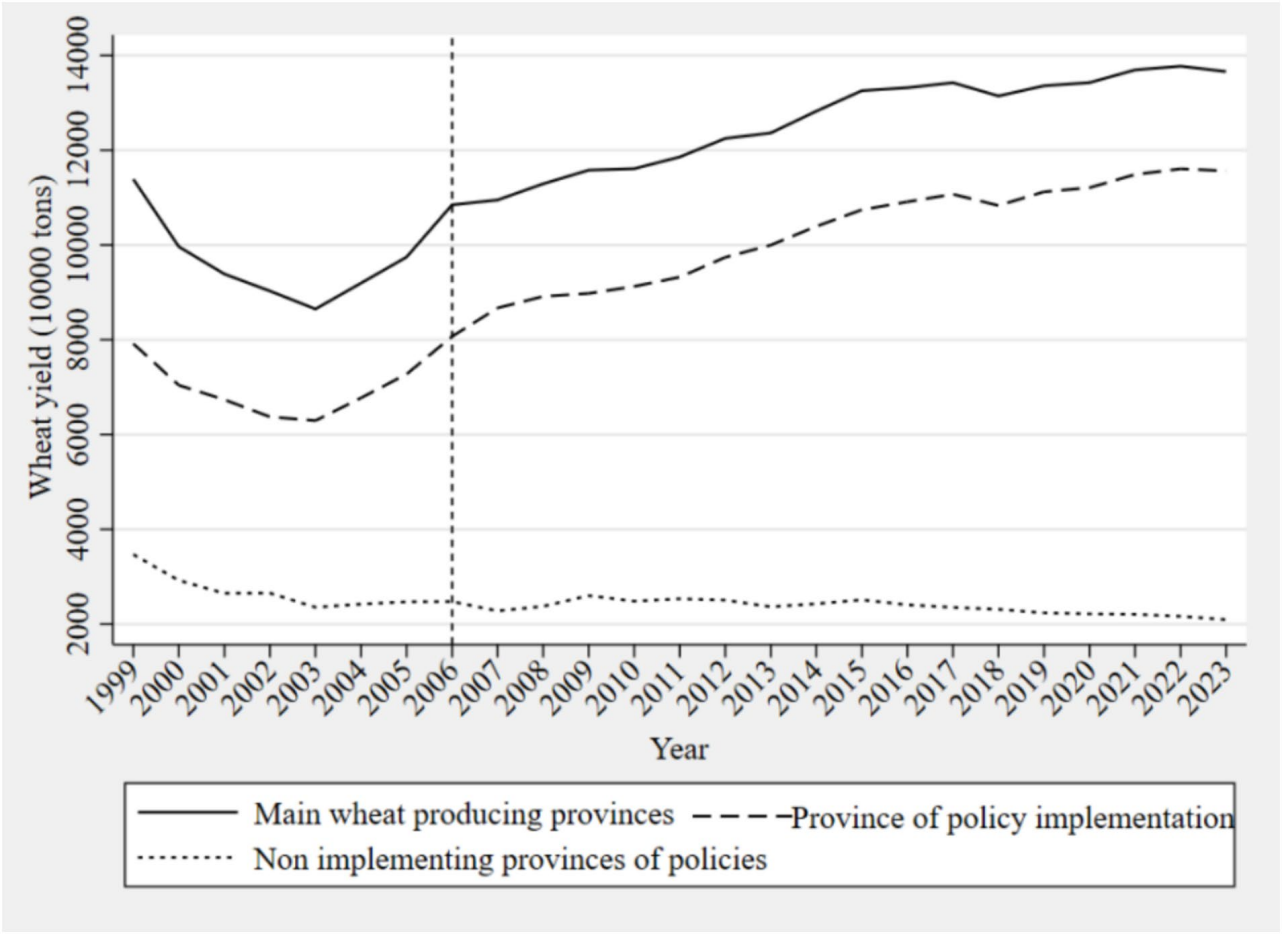
Trend chart of wheat production from 1999 to 2023.

Figure 5 illustrates the trend in wheat production from 1999 to 2023 in provinces that implemented the Minimum Purchase Price policy, non-implementing provinces, and major wheat-producing regions. According to the results, the overall increase in national wheat production has primarily relied on the growth in wheat output in provinces that implemented the Minimum Purchase Price policy. Furthermore, a clear divergence in trends is observed between policy-implementing and non-implementing provinces. Specifically, wheat production efficiency in the policy-implementing provinces shows a general upward trend, while in non-implementing provinces, it exhibits a downward trend, with the production gap between the two groups continuing to widen. This trend further substantiates the effectiveness of the policy and its impact on wheat production efficiency.

## 4 Analysis of the impact mechanisms of the Minimum Purchase Price policy on agricultural productivity

According to research on agricultural price intervention policies, both direct support policies affecting agricultural production and indirect subsidy policies influencing the production process affect various market participants through insurance, expectations, and wealth effects (26). Therefore, the mechanism through which the Minimum Purchase Price policy affects wheat production efficiency is as follows:

First, insurance safeguards the income of farmers who primarily depend on grain production, prevents low grain prices from harming farmers, and indirectly subsidizes grain prices to prevent high prices from burdening consumers (27). The widening income gap between agriculture and other industries places farmers at a lower income level, necessitating special protection for grain farmers to ensure food supply security (28, 29). In years of abundant harvests, grain prices decline when supply exceeds demand. Selling at the market equilibrium price under such conditions can lead to increased production without increased income, resulting in “low grain prices harming farmers.” During the wheat harvest season, if the market equilibrium (selling) price of wheat is higher than the minimum purchase price set by the National Development and Reform Commission and other relevant authorities, market participants purchase wheat at the actual selling price. If the selling price is lower than the minimum purchase price, the relevant purchasing departments and their designated entities buy wheat at the policy price to reduce the market circulation volume, thereby raising the selling price of wheat to a new equilibrium. Other market purchasers continue to buy at the actual market prices. Thus, the essence of the Minimum Purchase Price policy is government intervention in the supply of wheat to regulate market transaction prices, achieving a balance between supply and demand while ensuring farmers’ income.

Second, the expectation effect influences grain farmers’ production decisions by shaping price expectations. Current research on the effects of price-support policies on grain production focuses on farmers’ supply responses, transitioning from descriptive to empirical analyses. Early studies used static econometric methods, yielding overly simplistic conclusions that were detached from reality. In 1956, Nerlove introduced the Nerlove model, which considers the dynamic effects of farmers’ decision-making processes. This model assumes that economic agents learn from past mistakes and adjust future expectations based on the disparity between expected and actual prices. In China, where the household responsibility system dominates small-scale farming, farmers’ expectations of the minimum purchase price directly influence their planting decisions for the following year. Since 2016, when the Minimum Purchase Price policy broke the “only increase, no decrease” pattern, farmers primarily reliant on grain production have faced significant risks from falling grain prices, necessitating adjustments in the planting structure between cash crops and grain crops to maximize profits. Different farmers vary in resource endowments, management capabilities, dependence on agricultural production, and sensitivity to policies, resulting in diverse policy responsiveness (30–32). From the perspective of planting objectives, small-scale farmers, often part-time farmers, grow grains mainly for subsistence and are less affected by policy changes. Large-scale farmers, relying on grain for income, are more sensitive to policies that initially lease more land, but eventually experience decreased sensitivity due to the total cultivated land area, while small-scale rice farmers find it easier to adapt because of smaller land sizes, mitigating risks. Conversely, large-scale rice farmers face greater difficulties in adjusting their production structures because of their larger land areas and higher labor, material, and time costs. Additionally, various agricultural support policies can effectively mitigate losses from lower minimum purchase prices, thereby contributing to delayed adjustments. From the perspective of technology adoption, market intervention measures such as price support policies can generate benefits by accelerating the adoption of new technologies (33). Policy factors are the main determinants influencing the technology adoption rate among Chinese wheat farmers, such as preferential policies and agricultural subsidies, including the minimum purchase price policy (34). By stabilizing market prices, these policies provide farmers with higher income expectations, thereby incentivizing them to adopt new technologies to increase yield. For instance, after the implementation of the policy, wheat farmers in grain production functional areas such as the North China Plain have significantly improved mechanization levels and adoption rates of new varieties (35, 36). However, the effects of the policy show differences across different stages (37) In the early stages of policy implementation, the technology adoption rate increased significantly, particularly in key production areas (e.g., Henan and Shandong provinces). As the policy matured, the marginal returns to technology adoption decreased, and in some regions, farmers’ motivation to adopt new technologies weakened. Moreover, the policy’s effects vary according to farm characteristics. In areas with concentrated farming, large-scale farms, due to their abundant resources, are more likely to adopt new technologies, while small-scale farmers face financial and technological barriers, resulting in a lower adoption rate (38, 39). Therefore, future policy design should pay more attention to regional differences and farm characteristics to further enhance technology adoption rates and production efficiency.

Finally, the wealth effect of the Minimum Purchase Price policy is not particularly evident. The wealth effect involves direct subsidies to farmers through government transfer payments intended to increase their income levels and production investment capacity, thereby influencing their production decisions. Conversely, this could also dampen farmers’ enthusiasm for grain cultivation, leading to changes in the quantity and structure of grain production and potentially affecting production efficiency (39). However, farmers’ production behavior typically reflects a comprehensive evaluation of their current production conditions and the minimum purchase price, with minimal influence from external factors. Therefore, the wealth effect mechanism does not have a clear impact.

To examine how the effects of insurance coverage, price expectations, and wealth influence wheat production volatility, and to understand how these effects interact with fluctuations in yield and cultivated area, a detailed analysis can be conducted across three stages: the early, middle, and later phases of policy implementation (see Table 2). It was found that the insurance coverage effect plays a dominant role in the early stage of policy implementation, ensuring relatively stable production decisions among farmers. As the policy matures and the market-oriented reforms advance, the influence of this effect gradually diminishes. The price expectation effect, on the other hand, becomes increasingly important in the middle and later stages, particularly under the backdrop of market reforms and global grain price fluctuations, as farmers’ production decisions increasingly rely on market signals. The wealth effect influences production scale across all stages by affecting farmers’ income levels; however, its strength is significantly influenced by price volatility and policy adjustments. These three effects work together to shape wheat production volatility, with each effect dominating fluctuations in yield and cultivated area at different stages. In the early stage of policy implementation, the insurance coverage effect is most pronounced; in the middle stage, the price expectation effect strengthens; and in the later stage, both the wealth effect and the price expectation effect have a more substantial impact on production volatility.

**Table 2:** Comparative analysis of the early, middle, and later phases of policy implementation.

Effect	Early stage	Middle stage	later stage
Insurance coverage effect	Increase in farmers’ confidence, production area and output with government price guarantees	Decrease in reliance on policy, with production decisions increasingly dependent on market signals	Weakened insurance coverage effect, intensified market reforms, and farmers’ focus on market prices.
Price expectation effect	Farmers’ confidence in policy guarantees, expectation of price stability, and increased production area	Market volatility leading to farmers’ attention to price changes, with price expectations becoming the dominant factor	Increased farmers’ focus on market prices and heightened production volatility.
Wealth effect	Income growth led by policy guarantees, driving expansion of production scale	Stable price, weakened wealth effect and conservative farmer production decisions	Price volatility affecting farmers’ wealth status and significant fluctuations in production scale

## 5 Future developments

Based on the findings of this study, several important conclusions can be drawn. First, the implementation of the Minimum Purchase Price policy has not fully achieved balanced efficiency development across regions and production scales. While the policy has been effective in boosting food production in certain areas, its benefits have not been fully realized in others due to uneven resource allocation and the absence of a comprehensive market mechanism. As a result, production efficiency remains unbalanced. Therefore, it is essential that future policy implementation places greater emphasis on promoting balanced development at the macro level, ensuring that efficiency improvements are more evenly distributed across different regions. Second, the study identifies inefficiencies in the allocation of production factors as a key constraint on wheat production potential. Although wheat production in China has increased in recent years, the rate of improvement in production efficiency has not kept pace with the increase in input factors. This imbalance suggests that the allocation of key production factors, including land, capital, technology, and mechanization, remains suboptimal. In particular, the lack of advanced technology and mechanization has hindered further productivity gains. To address these challenges, it is crucial to accelerate the rational allocation and efficient utilization of these factors, with a particular focus on technological innovation and mechanization. Third, excessive policy intervention has distorted market mechanisms, impeding the development of a well-functioning market system. The MPP policy has led to a phenomenon of “policyization” in the food market, disrupting the positive interaction between upstream and downstream sectors of the agricultural value chain. To foster a more integrated industry, it is recommended that government intervention in the market be reduced and that policies promote the coordinated development of wheat production alongside related sectors, such as processing and distribution. This approach would contribute to the creation of a more efficient and sustainable agricultural ecosystem. Finally, the MPP policy mechanism exhibits certain limitations, necessitating its optimization. While the policy has effectively protected farmer incomes and increased food production, it has also led to market price distortions and rising production costs. As the economic environment evolves, these issues have become more pronounced, particularly with regard to the mismatch between grain prices and production costs. Therefore, it is essential that the policy mechanism be optimized to reduce excessive market intervention, ensuring that it can adapt to changing market conditions and achieve its maximum potential benefits.

Based on this study, the following policy recommendations are proposed:

### 5.1 Focus on the macro balance and coordinate equitable efficiency development

It is essential to consider the production differences among varieties and implement differentiated policies accordingly. Given the varying sensitivities of different agricultural products to policies, reforms and implementation should start with varieties that are less sensitive to policy changes. From the perspective of ensuring national food security, different functional types of agricultural products should be treated with differentiated policies, implementing the principle of “higher quality, better price,” and aligning prices with variety and quality grades, fully considering their functional uses to scientifically implement a reasonable Minimum Purchase Price policy. For example, wheat has three primary uses: staple food, agricultural raw materials, and animal feed. The production requirements for each use differ. Staple food and agricultural raw materials must meet high standards for safety, quality, nutrition, and health, whereas feed requires cost-effectiveness and large quantities. Therefore, policy formulations should consider factors such as production costs, profit margins, market supply, and the demand for different varieties. High-quality crop varieties should follow the principle of “higher quality, better price,” whereas lower-quality crops should adopt a strategy of low profit but high volume. This approach widens the price differences, thereby increasing income and stimulating the market. Specifically, high-quality grains, due to their superior characteristics (such as better taste, higher nutritional value, or greater processing yield), typically command higher market prices. These grains primarily target specific consumer segments willing to pay a premium for quality. Consequently, the pricing strategy for high-quality grains should account for both the rising production costs and the willingness of certain consumer groups to pay higher prices. In contrast, low-quality grains, which benefit from higher yields or lower production costs, are better suited to a high-volume, low-margin strategy. These grains generally cater to price-sensitive consumers or are used in industrial applications where quality requirements are lower. By setting lower prices for low-quality grains, producers can increase sales volumes to capture a larger market share, even though per-unit profit margins are relatively low. Through economies of scale, producers can compensate for lower profits by achieving higher sales volumes.

It is crucial to prioritize macroeconomic equilibrium and coordinate equitable efficiency development, taking into account the diversity of inter-provincial development and implementing spatial development policies. The strategy of prioritizing development in certain provinces has led to increasing disparities in production efficiency among China’s major grain-producing regions. Therefore, addressing the distribution and balance of the production system is crucial for the government as it focuses on food security and increasing wheat production. On the one hand, in high-efficiency wheat production areas, it is essential to develop appropriately scaled operations and strengthen government support. The potential of high-yield areas should be fully utilized by creating modern agricultural technology zones, high-quality farmland, advanced planting bases, and green “plant-breed” circular farms as long-term policy directions. Enhancing the role of cooperatives can further promote the transfer of advanced practices from leading to lagging regions, thereby coordinating overall balanced growth. On the other hand, in low-efficiency wheat production areas, policies should be more inclined toward guidance and support. These areas should learn from the production techniques and scientific methods of advanced regions and rationally allocate the production of economic grain crops to maximize benefits. Additionally, it is crucial to clarify the responsibilities of local governments and various departments, standardize agricultural investments, and prioritize agricultural and rural development, aiming to align with high-efficiency production areas to create a more balanced and efficient agricultural development framework, leverage policy tools to support farmers, optimize production, and ensure national food security.

### 5.2 Accelerate the allocation of production factors to unleash wheat production’s potential

First, regarding land resources, priority should be given to protecting China’s major grain production areas to prevent the conversion of arable land to non-grain uses. Additionally, through enhanced agricultural infrastructure construction and the establishment of high-standard farmland, intensive management and specialized production should be promoted to ensure more stable production under the Minimum Purchase Price policy. The income security provided by the Minimum Purchase Price policy offers farmers stable market expectations. Therefore, optimizing the land transfer system and ensuring farmers’ contractual rights will not only improve land use efficiency but also promote the sustainable development of grain production. This is particularly important in regions with low production efficiency. By strengthening scientific crop rotation and improving cultivation methods, land productivity can be intensified. At the same time, reinforcing policy support, such as subsidies for quality seeds and agricultural machinery, can mitigate the potential negative impact of the Minimum Purchase Price policy on technological progress and facilitate technological upgrading.

Second, in terms of human resources, the stable prices guaranteed by the Minimum Purchase Price policy ensure farmers’ income but may also reduce their reliance on technological innovation. Therefore, it is essential to enhance knowledge and technology training for agricultural workers, improving farmers’ professionalism to ensure that agricultural production does not solely depend on price protection but relies on technological advancements to enhance efficiency. By increasing the degree of organization among farmers, agricultural production can be further specialized and modernized, reducing excessive dependence on policies. Particularly in the context of labor shortages and an aging workforce, it is important to promote agricultural modernization and optimize the labor structure, encouraging the return of younger laborers to the agricultural sector.

Finally, in terms of capital investment, the income security provided by the Minimum Purchase Price policy encourages farmers to plan their production investments over the long term. However, to avoid excessive reliance on price guarantees, the capital investment structure for wheat production should be optimized to prevent resource waste and environmental pollution. In low-efficiency areas, accelerating the promotion of agricultural mechanization and utilizing policy support to reduce the cost of mechanization can improve production efficiency. Furthermore, improving irrigation infrastructure, especially the widespread adoption of water-saving irrigation systems, will unlock production potential. Additionally, the rational use of chemical inputs, such as fertilizers and pesticides, should be promoted to prevent overuse that may lead to environmental pollution, ensuring sustainable agricultural development.

### 5.3 Create a favorable external social environment for integrated industrial development

The process of urban–rural integration can lead to changes in the development levels of urban and rural areas, primarily reflected in economic and cultural development. From the perspective of economic development, urbanization and industrialization have accelerated economic growth. This process creates conditions conducive to enhancing agricultural infrastructure, investing in technological research and development, and optimizing the production resource environment, thereby facilitating the specialization of grain production. From the perspective of cultural development, improving the overall level of education may lead to the transfer of labor to non-agricultural employment, which helps cultivate new types of agricultural production and management entities. Governments at all levels should provide targeted training and guidance to key grain-growing entities. Additionally, offering various technical support and favorable agricultural policies can help establish new, specialized grain production entities. Collectively, these measures can create a supportive external environment that fosters the integration of agricultural and industrial development, promoting both economic growth and the modernization of agricultural practices.

### 5.4 Optimize the Minimum Purchase Price policy mechanism to fully utilize policy advantages

Reforming the “one-size-fits-all” unified pricing method is essential. Utilizing modern information technology and establishing platforms that connect farmers, purchasing enterprises, and third-party financing institutions can increase information transparency. This would provide farmers with more data for production forecasts, thereby reducing their reliance on state-set prices. Transitioning development strategies to a “high quality, better price” sales principle based on subdivided grain varieties and quality grades is key to reducing policy execution costs, creating a healthy grain market circulation mechanism, and alleviating state policy grain storage pressures. Focusing on financial support for technological innovation in agricultural production is crucial for advancing agricultural productivity. This approach prevents the diminishing marginal returns caused by the weak execution of the Minimum Purchase Price policy, ensuring that food security measures are effectively implemented. Additionally, motivating farmers to engage in grain cultivation through enhanced technical training and promoting rational crop rotation are essential. Actively leveraging market mechanisms, clarifying the roles and responsibilities of different departments, and improving policy implementation efficacy will help stabilize the primary sector. Given that the Minimum Purchase Price policy has been in place for a relatively short time, accurately assessing whether the policy goals have been met is challenging. Therefore, it is necessary to adapt policies to local conditions and provide region-specific guidance based on local farmland productivity. The continuous adjustment of the subsidy mechanism ensures maximum effectiveness. Stabilizing price fluctuations facilitates price guidance and discovery in both the domestic and international markets. Frequent linkages between the domestic and international markets affect the effectiveness of the Minimum Purchase Price policy. Price differences between domestic and international grain markets influence spot market price fluctuations and have a spillover effect on futures market volatility. Moreover, fluctuations in the futures market are closely related to foreign trade policies, further weakening price formation factors and encouraging farmers to autonomously adjust planting structures based on market demand, thereby minimizing government intervention to the greatest extent possible.

## 6 Conclusion

This study addressed the current challenges in China’s staple grain production efficiency by examining the impact of the Minimum Purchase Price policy on wheat production efficiency from the perspective of price support. First, the relevant literature on price intervention policies was collected, summarized, and organized, covering aspects such as the implementation effects of the Minimum Purchase Price policy, its social welfare effects, pricing studies, and comparisons of wheat production efficiency both domestically and internationally. Second, this study considered the current development of wheat production efficiency to explore the mechanisms through which the Minimum Purchase Price policy affects wheat production efficiency. Finally, policy recommendations were proposed.

In the review section, the analysis systematically examines the impact of the Minimum Purchase Price policy on wheat production efficiency amidst the current sluggish growth in China’s grain output. The current state of wheat production efficiency in China is described, and the underlying mechanisms are explored, with the aim of further improving wheat production efficiency. This study also proposed methods for optimizing and improving the evaluation system of current agricultural support policies, which will benefit the macro-control of grain production resource allocation, fully utilize regional production advantages to increase grain output, aid rural revitalization, and help farmers increase their income levels.

Furthermore, from an international perspective, this study provides a comparative analysis of China’s Minimum Purchase Price policy (MPP), the European Union’s Common Agricultural policy (CAP), and U.S. agricultural subsidies (primarily agricultural income support programs, such as subsidies and price supports), focusing on their impacts on agricultural production efficiency, market distortions, and farmers’ income, as detailed in Table 3. These three agricultural policies share many similarities in terms of income protection, market intervention, and promoting agricultural production, as they all involve some form of government intervention to ensure farmers’ income and market stability. However, there are significant differences in their emphasis: China focuses on food security and short-term income guarantees, the European Union emphasizes sustainable agricultural development and environmental protection while supporting small-scale farmers, and the United States prioritizes increasing agricultural production efficiency and market competitiveness, with subsidies concentrated on large-scale agricultural production. These policies reflect the different strategies adopted by each country based on its agricultural development status and priority needs. Although their goals are similar, the implementation methods and areas of focus vary.

**Table 3:** Comparative analysis of China’s Minimum Purchase Price policy, the European Union’s Common Agricultural policy, and U.S. agricultural subsidies.

Policy	Agricultural production efficiency	Market distortion	Farmer income	Common points	Focus
China’s Minimum Purchase Price policy	Short-term yield growth assurance, long-term efficiency limitations	Price distortion and resource misallocation	Minimum income guarantee, with potential limitation on long-term income growth	Income guarantee, market intervention, price support, agricultural production promotion	Food security and income stabilization focus, short-term income guarantee for farmers
EU Common Agricultural policy	Limited improvement in production efficiency, shifting toward sustainable agriculture	Minimal market distortion, primarily subsidy allocation issues and over-reliance on large farms	Stable income provision, with potential disproportionate benefit to large farms and short-term income fluctuations	Income guarantee, market intervention, price support, agricultural production promotion	Sustainability and environmental focus, small farm livelihood support
U.S. agricultural subsidies	Higher production efficiency, especially in large-scale farming	Risk of over-reliance on monoculture and production surplus	Income protection, particularly against market fluctuations and disasters, with potential hindrance to innovation	Income guarantee, market intervention, price support, agricultural production promotion	Focus on increasing production efficiency and market competitiveness, large-scale production and efficient agriculture

## References

[1] Anderson K. Krueger, Schiff, and Valdes revisited: agricultural price and trade policy reform in developing countries since 1960. Appl Econ Perspect Policy. (2010) 32:195–231. doi: 10.1093/aepp/ppq005

[2] Attanasio O Di Maro V Lechene V Phillips D. Welfare consequences of food prices increases: evidence from rural Mexico. J Dev Econ. (2013) 104:136–51.

[3] Wang W Wei L. Impacts of agricultural price support policy on price variability and welfare: evidence from China’s soybean market. Agric Econ. (2021) 52:3–17. doi: 10.1111/agec.12603

[4] Swaminathan MS Vepa SS. How can India help prevent food price volatility? IDS Bull. (2012) 43:84–91. doi: 10.1111/j.1759-5436.2012.00350.x

[5] Gohin A Zheng Y. Reforming the European common agricultural policy: from price & income support to risk management. J Policy Model. (2020) 42:712–27. doi: 10.1016/j.jpolmod.2020.02.008

[6] Jha S Srinivasan PV. Grain price stabilization in India: evaluation of policy alternatives. Agric Econ. (1999) 21:93–108. doi: 10.1111/j.1574-0862.1999.tb00586.x

[7] Brooks J. Policy coherence and food security: the effects of OECD countries’ agricultural policies. Food Policy. (2014) 44:88–94. doi: 10.1016/j.foodpol.2013.10.006

[8] Xie H Wang B. An empirical analysis of the impact of agricultural product price fluctuations on China’s grain yield. Sustain For. (2017) 9:906. doi: 10.3390/su9060906

[9] Tao Y. Study on the influence of economic policy uncertainty on food price volatility Qingdao (2020). doi: 10.25236/edssr.2020.126

[10] Covaci S Sojková Z. Investigation of wheat efficiency and productivity development in Slovakia. Agric Econ. (2006) 64:368.

[11] Balcombe K Fraser I Rahman M Smith L. Examining the technical efficiency of rice producers in Bangladesh. J Int Dev. (2007) 19:1–16. doi: 10.1002/jid.1284

[12] Rakotoarisoa MA. The impact of agricultural policy distortions on the productivity gap: evidence from rice production. Food Policy. (2010) 36:147–57. doi: 10.1016/j.foodpol.2010.10.004

[13] El-Rasoul AA Ramadan AM El-Seify E Shehab SM. Total factor productivity and environmental efficiency of the most important cereals crops in Egypt. Asian J Econ Busi Account. (2020) 15:1–17. doi: 10.9734/ajeba/2020/v15i430218

[14] Mahmood N Arshad M Kächele H Ullah A Müller K. Economic efficiency of rainfed wheat farmers under changing climate: evidence from Pakistan. Environ Sci Pollut Res Int. (2020) 27:34453–67. doi: 10.1007/s11356-020-09673-5 32557029

[15] Aslam MS Xue PH Bashir S Alfakhri Y Nurunnabi M Nguyen VC. Assessment of rice and wheat production efficiency based on data envelopment analysis. Environ Sci Pollut Res. (2021) 28:38522–34. doi: 10.1007/s11356-021-12892-z, PMID: 33738743

[16] Rachman H Tri H Wulan SD. Technical efficiency among agricultural households and determinants of food security in East Java, Indonesia. Sci Rep. (2021) 11:4141. doi: 10.1038/s41598-021-83670-7, PMID: 33603009 PMC7893039

[17] Krasachat W. Measurement of technical efficiency in Thai agricultural production [Z]. Bangkok: Kasetsart University (2000).

[18] Loke PF KotzÃ E Du Preez CC Twigge L. Long-term effects of wheat production management practices on some carbon fractions of a semiarid Plinthustalfs. Soil Res. (2018) 56:601–14. doi: 10.1071/SR18050

[19] Awal A-R Awudu A. Do farmer groups impact on farm yield and efficiency of smallholder farmers? Evidence from rice farmers in northern Ghana. Food Policy. (2018) 81:95–105. doi: 10.1016/j.foodpol.2018.10.007

[20] Dias Bernardes Gil J Reidsma P Giller K Todman L Whitmore A Van Ittersum M. Sustainable development goal 2: improved targets and indicators for agriculture and food security. Ambio. (2019) 48:685–98. doi: 10.1007/s13280-018-1101-4, PMID: 30267284 PMC6509081

[21] Costanzo A Bàrberi P. Functional agrobiodiversity and agroecosystem services in sustainable wheat production. A review. Agron Sustain Dev. (2014) 34:327–48. doi: 10.1007/s13593-013-0178-1

[22] Morteza T Soheili-Fard F Rohani A Chen G Yildizhan H. Life cycle assessment to compare the environmental impacts of different wheat production systems. J Clean Prod. (2018) 197:195–207. doi: 10.1016/j.jclepro.2018.06.173

[23] Gliessman S. Putting food into agricultural policy. Agroecol Sustain Food Syst. (2019) 43:603–604. doi: 10.1080/21683565.2019.1628167

[24] Liu Z Liang H Pu D Xie F Zhang E Zhou Q. How does the control of grain purchase price affect the sustainability of the national grain industry? One empirical study from China. Sustain For. (2020) 12:2102–2. doi: 10.3390/su12052102

[25] Lankoski J Thiem A. Linkages between agricultural policies, productivity and environmental sustainability. Ecol Econ. (2020) 178:106809. doi: 10.1016/j.ecolecon.2020.106809

[26] Anang BT Bäckman S Rezitis A. Production technology and technical efficiency: irrigated and rain-fed rice farms in northern Ghana. Eurasian Econ Rev. (2017) 7:95–113. doi: 10.1007/s40822-016-0060-y

[27] Li J Chen J Liu H. Sustainable agricultural total factor productivity and its spatial relationship with urbanization in China. Sustain For. (2021) 13:6773. doi: 10.3390/su13126773

[28] Ge D Long H Zhang Y Tu S. Analysis of the coupled relationship between grain yields and agricultural labor changes in China. J Geogr Sci. (2018) 28:93–108. doi: 10.1007/s11442-018-1461-5

[29] Wu P Zhao G Liu F Ahmad S Fan T Li S. Agronomic system for stabilizing wheat yields and enhancing the sustainable utilization of soil: a 12-year in-situ rotation study in a semi-arid agro-ecosystem. J Clean Prod. (2021) 329:129768. doi: 10.1016/j.jclepro.2021.129768

[30] Rahman S Hasan MK. Impact of environmental production conditions on productivity and efficiency: a case study of wheat farmers in Bangladesh. J Environ Manag. (2008) 88:1495–504. doi: 10.1016/j.jenvman.2007.07.019, PMID: 17764818

[31] Le Gouis J Oury F-X Charmet G. How changes in climate and agricultural practices influenced wheat production in Western Europe. J Cereal Sci. (2020) 93:102960. doi: 10.1016/j.jcs.2020.102960

[32] Kashyap D Agarwal T. Temporal trends of climatic variables and water footprint of rice and wheat production in Punjab, India from 1986 to 2017. J Water Clim Change. (2020) 12:1203–19. doi: 10.2166/wcc.2020.093

[33] Miller T Tolley G. Technology adoption and agricultural price policy. Am J Agric Econ. (1989) 71:847–57. doi: 10.2307/1242662

[34] Kurkalova L Kling C Zhao J. Green subsidies in agriculture: estimating the adoption costs of conservation tillage from observed behavior. Can J Agric Econ. (2006) 54:247–67. doi: 10.1111/j.1744-7976.2006.00048.x

[35] Meng M Zhang W Zhu X Shi Q. Agricultural mechanization and rural worker mobility: evidence from the agricultural machinery purchase subsidies programme in China. Econ Model. (2024) 139:106784. doi: 10.1016/j.econmod.2024.106784

[36] Li T Yang F Zhang H Lin Q. The effectiveness and mechanisms of China’s grain support policies in relation to grain yield—an evaluation of a wide range of policies. Food Secur. (2025) 14:267. doi: 10.3390/foods14020267, PMID: 39856933 PMC11764825

[37] Ruzzante S Labarta R Bilton A. Adoption of agricultural technology in the developing world: a meta-analysis of the empirical literature. World Dev. (2021) 146:105599. doi: 10.1016/j.worlddev.2021.105599 PMC8479612 34621923

[38] Xu D Liu Y Li Y Liu S Liu G. Effect of farmland scale on agricultural green production technology adoption: evidence from rice farmers in Jiangsu province China. Land Use Policy. (2024) 147:107381. doi: 10.1016/j.landusepol.2024.107381

[39] Zhang J Jabbar A Li X. How does China’s agricultural subsidy policy drive more commercially productive small farmers? The role of farmland scale, labor supply, and cropping structural change. Land. (2024) 13:2058. doi: 10.3390/land13122058

[40] Lu J Xiang Y Fan J Zhang F Hu T. Sustainable high grain yield, nitrogen use efficiency and water productivity can be achieved in wheat-maize rotation system by changing irrigation and fertilization strategy. Agric Water Manag. (2021) 258:107177. doi: 10.1016/j.agwat.2021.107177

[41] Simmons AT Cowie AL Brock PM. Climate change mitigation for Australian wheat production. Sci Total Environ. (2020) 725:138260. doi: 10.1016/j.scitotenv.2020.138260 32298879

